# Are you a good mimic? Neuro-acoustic signatures for speech imitation ability

**DOI:** 10.3389/fpsyg.2013.00782

**Published:** 2013-10-22

**Authors:** Susanne M. Reiterer, Xiaochen Hu, T. A. Sumathi, Nandini C. Singh

**Affiliations:** ^1^Unit for Language Learning and Teaching Research, Faculty of Philological and Cultural Studies, University of ViennaVienna, Austria; ^2^Centre for Integrative Neuroscience and Hertie Institute for Clinical Brain Research, University Clinic TübingenTübingen, Germany; ^3^Neurology Department (Hertie, CIN), Hertie Institute for Clinical Brain Research, University of TübingenTübingen, Germany; ^4^Department of Psychiatry, University of BonnBonn, Germany; ^5^National Brain Research CentreManesar, India

**Keywords:** speech imitation, second language acquisition, aptitude, fMRI, spectral analysis, Fourier transform, individual differences, multilingualism

## Abstract

We investigated individual differences in speech imitation ability in late bilinguals using a neuro-acoustic approach. One hundred and thirty-eight German-English bilinguals matched on various behavioral measures were tested for “speech imitation ability” in a foreign language, Hindi, and categorized into “high” and “low ability” groups. Brain activations and speech recordings were obtained from 26 participants from the two extreme groups as they performed a functional neuroimaging experiment which required them to “imitate” sentences in three conditions: (A) German, (B) English, and (C) German with fake English accent. We used recently developed novel acoustic analysis, namely the “articulation space” as a metric to compare speech imitation abilities of the two groups. Across all three conditions, direct comparisons between the two groups, revealed brain activations (FWE corrected, *p* < 0.05) that were more widespread with significantly higher peak activity in the left supramarginal gyrus and postcentral areas for the low ability group. The high ability group, on the other hand showed significantly larger articulation space in all three conditions. In addition, articulation space also correlated positively with imitation ability (Pearson's *r* = 0.7, *p* < 0.01). Our results suggest that an expanded articulation space for high ability individuals allows access to a larger repertoire of sounds, thereby providing skilled imitators greater flexibility in pronunciation and language learning.

## Introduction

Speech sound imitation is a pivotal learning mechanism for humans. Vocal imitation provides a basis for acquisition of both languages and musical systems (Fitch, 2010). Yet individuals differ greatly in their aptitude, ability, and success in sound imitation learning (Golestani et al., 2002, 2011; Dogil and Reiterer, 2009; Reiterer et al., 2011). This is especially evident when it comes to the acquisition of a second language sound system, with diverse shades of foreign accent being left as audible traces for the listener. Some people, on the other hand, are adept at vocal imitation and make a living mimicking dialects, speech characteristics, and foreign accents.

Although the origins of differences in imitation ability—whether neurobiological, genetic, psycho-cognitive, social or environmental are still unknown, recent neuroscientific research has suggested brain structural and functional differences as a partial explanation (e.g., Golestani et al., 2007; Golestani and Pallier, 2007; Reiterer et al., 2011). Anatomical predispositions in the brain have been proposed to explain why some individuals have better speech sound imitation or auditory discrimination skills (Golestani and Pallier, 2007; Wong et al., 2008; Harris et al., 2009; Golestani et al., 2011). While the direction of correlation between skill and level of brain activation continues to be hotly debated, neuroimaging experiments have consistently reported, that greater amounts of brain activation accompany poor imitation skill. This phenomenon of “cortical efficiency/effort” (Reiterer et al., 2005a,b; Prat et al., 2007) suggests that speakers with higher speech imitation ability show reduced brain activation in brain networks related to pronunciation, phonemic awareness, articulation, phonological processing, sound imitation, and auditory working memory (Reiterer et al., 2011).

An interesting combination of acoustic and neurophysiological methods has been applied to characterize speech discrimination ability in animals. Using spectrograms and neural activity in the form of spike timing and firing rate, the performance success of consonant discrimination ability could be predicted by neural activity in the primary auditory cortex of rats (Engineer et al., 2008).

Along similar lines, but in the domain of individual differences of voice recognition, forensic phonetics has used acoustic biometrics to identify individual speakers wherein time-frequency spectral signal analysis has been used to identify phonetic, phonation or voice characteristics in speech. (Arsikere et al., 2010; Disner et al., 2010; Belin et al., 2011; Tiwari and Tiwari, 2012).

Past studies have suggested that second language speakers or late bilinguals have difficulties imitating foreign sounds and phonemes as reflected in their phonetic performance (Piske et al., 2002), due to a so-called phonological filtering called “interference,” based on their intense experience with the mother tongue (L1). However, this widely held belief has recently been challenged by a less radical view that attributes these differences to individual differences in language aptitude (Abrahamsson and Hyltenstam, 2008; Reiterer et al., 2011; Hu et al., 2013). This view has also proposed that a certain proportion of individuals is able to overcome this filter, and therefore shows lifelong adaptive imitative articulation behavior, a behavioral characteristic in the neuro-motor-acoustic domain called “vocal flexibility.”

Given these observable differences in the neuro-biological and acoustic-phonetic domain, we hypothesized that it should be possible to find quantitative bio-markers to measure differences in speech imitation ability biometrically that correlate with acoustic markers. Our objective was to combine the acoustic-phonetic and neuro-imaging domains to characterize good and bad pronunciation performance which reflected the lower and higher quantiles (second and third SD above and below the mean) at the ends of a normally distributed curve of individual differences in speech imitation capacity. We hypothesized that lower ability speech imitators would engage more widespread, diffuse neural activation around articulation-relevant areas in the brain and that acoustic analyses would be capable of capturing differences in segmental and suprasegmental speech articulation patterns as a function of individual differences in speech imitation ability.

## Methods

### Participants and behavioral experiments

One hundred and thirty-eight German-English second language learners (or, late bilinguals/polyglots) with varying degrees of proficiency in English, matched for age, handedness, gender, educational status, mother tongue, age of onset of second language acquisition (AOA), linguistic experience, neurological status, verbal and non-verbal IQ were randomly recruited for the study. There were no “true,” “equal” or early bilinguals (those with AOA before puberty) included in the study. Participants were randomly recruited from the urban catchment areas surrounding the universities of Tübingen and Stuttgart. This large pre-search pool was investigated closely with respect to speech imitation/speech production abilities to characterize the participants' behavioral performance in articulation ability to be better able to select participants for the further examinations. Their mean age was 25.9 years (*SD* ± 5.2), 90% right-handers, 85 females/53 males, mixed fields of study backgrounds from natural sciences, business to humanities and languages, mother tongue German, AOA: 10.5 years (*SD* ± 0.8), 50% language study background, no neurological disorders. They all knew at least one foreign language, which was English, 24% knew only one L2 (English), 30% knew two L2s, 22% knew three, 17.5% knew four, 3.5% five, 2% six, and 1% nine foreign languages. All participants together knew 27 different foreign languages to varying degrees including very low levels of proficiency for some of the mentioned languages: English, French, Italian, Spanish, Dutch, Greek, Portuguese, Hungarian, Turkish, Polish, Russian, Czech, Slowenian, Rumanian, Norwegian, Swedish, Danish, Finnish, Hebrew, Arabic, Persian, Korean, Japanese, Chinese, Africans, Esperanto, and Latin. Mean number of foreign languages was 2.5, minimum was 0 (additional to English) and maximum number was 9. All of these additional languages were acquired between age 10 and 35 at various ages within this range. All participants gave informed written consent for their participation in the study. The study was approved by the local Ethics Committee and was in accordance with the Helsinki declaration.

The mean exposure to formal school instruction in L2 English was: 9.9 years (±2.3). Their verbal (Lehrl et al., 1995) and nonverbal IQ (Raven et al., 1998) scores were in the normal to advanced range (between 110 and 140) corresponding to their educational background. In order to elicit speech imitation aptitude, we used indirect (imitation from memory) and direct imitation tasks as described by Flege and Hammond (1982) which required the volunteers either to fake a familiar foreign accent from long term memory (indirect or “delayed” imitation) or from acoustic working memory (direct imitation).

The experiments were carried out in subsequent steps. First of all, the large pre-search pool of 138 was investigated with linguistic and psycho-cognitive testing and speech imitating ratings were performed. After these procedures (~0.5–1 year afterwards) participants of this pre-search pool were asked for willingness to participate in the fMRI experiments. 53 participants, distributed over all “imitation ability” groups were willing to participate again in the MR experiments. 13 needed to be excluded because of various reasons including MR safety issues (tattoos, dental retainers, left handedness), so that 40 participants could be scanned and presented with our fMRI task “experiment 2,” described below under “behavioral experiment 2 (“sentence reading and accent faking”).

#### Behavioral experiment 1 (“Hindi imitation score”)

To characterize their basic ability of foreign sound imitation, the 138 participants were recorded in a sound-proof room while they performed different speech imitation, pronunciation or reading tasks in the languages German (L1), English (L2), and Hindi (L0 for unknown language). For details of the different task types and elicitation techniques see Jilka (2009).

To avoid the influence of language experience and to elicit the purest imitation capacity possible, the participants were exposed to speech material in Hindi, a language they had never heard before (Flege and Hammond, 1982). They had to repeat sentences spoken by a model Hindi speaker (direct imitation). The imitations were based on 4 Hindi sentences of different length and phonetic complexity (7/7/9/11 syllables long) which had to be repeated immediately after presentation. We repeated the stimulus sentences three times before imitation, to ensure sufficient exposure for reproduction. Evaluation of the quality of speech imitation of these stimuli was performed in India with online blind native speaker ratings of the participants' speech productions. The raters were naïve with regard to phonetic background and whom they rated. They were instructed to judge whether the sample they were listening to could be spoken by a native speaker of Hindi or not. To ensure the quality of the evaluation procedure, we randomly inserted recordings of Indian native speakers (*N* = 18) into the database. The speech samples were presented in random order. For the intuitive rating scale (Jilka, 2009) we used a rating bar which ranged from 10 to 0 (highest to lowest representation of native-speaker-likeness). 30 gender-balanced Indian native speakers in India rated all the samples online using earphones. The mean scores of all of the final 30 raters (after careful exclusion of low-fidelity raters) on the 4 sentences in the mean were used to form a basic imitation ability score (the “Hindi score”). More details about the Hindi imitation scoring are also provided in the results section Behavioral Results.

#### Behavioral experiment 2 (“sentence reading and accent faking”)

Inside of the fMRI scanner (reported below) we performed further indirect imitation tasks, namely sentence reading tasks (imitation from memory). 26 participants belonging to the highest and lowest ability groups according to the behavioral experiment 1 (Hindi imitation scoring) were scanned and recorded in the scanner on sentence reading tasks in their L1 German, L2 English, and “faking an accent” (faking L2 in L1). We chose this task to be performed in the scanner, because it was described in the literature as a task which discriminates well between high and low aptitude second language learners in accent imitation because phonemic awareness is especially taxed in the case of the imitations from memory (Flege and Hammond, 1982). The task is described in more detail in Neuroimaging Tasks.

### Neuroimaging analyses

#### Neuroimaging tasks

Based on the ratings of the Hindi imitation task of our large pre-selection study, participants were categorized along a continuum of the Hindi imitation ability score. From this overall pool of 138 subjects we re-invited a similarly (normally) distributed subsample to our fMRI experiments. 40 participants were scanned. For the fMRI group vs. group analysis (*N* = 26) we selected the two extreme groups within the second and third SD below and above the mean, resulting in 13 participants each group. Details for the high ability group: 9 males/4 females, mean age was 28.38 years (*SD* ± 5.0), age of onset of L2 English: 10.38 years (*SD* ± 0.9), mean number of second languages spoken: 2.38 (*SD* ± 1.1), mixed study background, Hindi score: 6.4 (*SD* ± 0.7). Details for the low ability group: 8 males/5 females, mean age was 25.46 years (*SD* ± 5.0), age of onset of L2 English: 10.69 years (*SD* ± 0.9), mean number of second languages spoken: 2.23 (*SD* ± 1.3), mixed study background, Hindi score: 3.6 (*SD* ± 0.7). The two groups were significantly different with regard to the Hindi score (*p* = 0.000^**^), but no other significant group differences were found in these behavioral background parameters.

We scanned these already well described participants (see also Participants and Behavioral Experiments) during overt, microphone-monitored speech production (sentence reading). The monitored utterances were quality controlled in two ways: once online during the in-scanner task and once offline for omissions and error rates. Using an event-related, sparse-sampling fMRI paradigm, we administered an overt sentence reading task (30 min), which was subdivided into the three sub-conditions: (A) reading aloud German sentences (L1), (B) reading aloud English sentences (L2) and (C) reading German sentences with a fake English accent (any variety). This task required phonemic awareness of foreign accented speech characteristics. We chose this task to be performed in the scanner, because it was described in the literature as a task which discriminates well between high and low aptitude second language learners in accent imitation because phonemic awareness is especially taxed in the case of the imitations from memory (Flege and Hammond, 1982). Mean sentence production duration was 3 s. The sentences were presented in the center of the screen and condition (C) was signaled by a British flag symbol above the sentence. The 75 total stimuli (25 per condition) were 11-syllables long and matched for semantic content. Participants were instructed to start reading as soon as the sentence appeared. For acquisition, a sparse sampling paradigm was used (*TR* = 12 s, *TA* = 3 s, pause = 9 s) with sentences presented and read during the scanner pauses. Stimuli were randomized for time-onsets and order of presentation. Inter-stimulus baseline trials were inserted alternatingly every second *TR* accompanied by fixating a white cross on black screen. The produced speech was recorded by a commercially available optical MR-microphone. Before the start of the fMRI scanning session subjects were familiarized with sample stimuli. Examples of each condition are provided below.

- “Die gute Mensa begeistert uns alle” (L1 German) (English translation: we all like excited about the good cafeteria)
- “The mechanic repaired cars in the garage” (L2 English)
- “Der Professor präsentiert das Resultat”(L1 with fake English accent) (English translation: the professor presents the result)

#### MR image acquisition

A Siemens Vision 1.5 T scanner was used. For functional imaging (fMRI) of the blood oxygen level dependent (BOLD) signal, we used an echo planar imaging (EPI) Gradient Echo sequence with sparse sampling method set at the following parameters: *TR* = 12 s, *TA* = 3 s, delay in *TR* (pause) = 9 s, *TE* = 48 ms, slice number = 36 transversal, Flip angle: 90°, Slice thickness = 3 mm + 1 mm gap, Voxel Size: 3 × 3 × 4 mm^3^, FoV = 192 × 192 × 143 mm^3^, ma-trix = 64 × 64. EPI images were acquired in transversal orientation. Acquisition was interleaved and descending.

For structural (anatomical) image acquisition, structural MRI scans of all subjects were performed on the same scanner, using a high resolution T1-weighted MDEFT sequence (Modified Driven-Equilibrium Fourier Transform), scantime = 12 min, rep-etition time (*TR*) = 7.92 ms; echotime (*TE*) = 2.48 ms; inversion time (*TI*) = 910 ms; *FA* = 16°; voxelsize: 1 mm × 1 mm × 1 mm, FoV = 176 mm × 256 mm × 256 mm, slices per slab = 176 sagittal, matrix = 256 × 256, acquisition orientation: transversal. An eight-channel headcoil was used.

The voice recordings from the scanner were subjected to the digital spectral analysis (acoustic measurement) described in the following sections.

#### fMRI statistical analysis

FMRI images were analyzed using the free software packet SPM5 (Statistic Parametric Mapping).

Data pre-processing: each fMRI data set underwent spatial realignment by aligning the first scan from each session with the first scan of the first session and aligning the images within sessions with the first image of a particular session. The realigned data were spatially normalized to the standard Montreal neurological institute (MNI) T1 template, with the coregistered individual T1 image as a reference. Volumes were resliced to a voxel size of 3 mm × 3 mm × 3 mm, motion corrected and spatially smoothed using a10-mm full-width-at-half-maximum Gaussian kernel and prepared for later random effects analyses. Temporal correction (slice timing) was not applicable because the sparse sampling acquisition mode was adopted here.

At the first level, design matrices of individual general linear models incorporated three regressors of language type (German, English, German with fake accent) for the task sentence reading task. Additional six regressors of movement parameters were added as well (roll, pitch, yaw, *x, y*, and *z*). Regressors were defined with onsets at the time of appearance of the corresponding event and convolved with the canonical hemodynamic response function. At the second level, group analysis was performed using analysis of variance (ANOVA), with one between subject factor “ability group” (high vs. low ability group) and one within-subjects factor “language type” (L1, L2, LAcc). Main effects for group and language type and the interaction effect of group by language type were calculated separately for each session. A statistical threshold of *p* < 0.05 (whole brain cluster level correction for multiple comparisons) was obtained. Results were overlaid on the mean anatomical image of the group (*N* = 26) and for the activation areas to be displayed we used a cluster extent threshold of 400 voxels.

### Acoustic analysis (modulation spectrum analysis)

The speech imitation recordings of the reading tasks in the scanner were subjected to detailed spectral analysis. The spectral analysis involved setting up the speech modulation spectrum (SMS), which a probability distribution of the different spectral and temporal modulations in a vocal utterance. It is obtained by calculating the two-dimensional Fourier transform of the autocorrelation matrix of the amplitude envelope of the vocal utterance in its spectrographic representation (Singh and Theunissen, 2003; Singh and Singh, 2008; Sullivan et al., 2013). In order to construct the SMS for each individual, all recordings were first manually examined by listening to the recorded speech. Recordings from the MRI scanner were cleaned in order to remove scanner noise using Goldwave (vesion 5.10). Speech waveform files that were of very low recording quality were excluded from this study. Amplitude waveforms were normalized to -18 dB. Finally, all analyzed speech recordings for each speaker were composed of 25 sentences per condition, sentence length was 3 s. This process was carried out for each condition of the fMRI task: reading German, English, and German with fake English accent (conditions A, B, C). Using custom written code in Matlab the SMS for each individual for each imitation condition was constructed using the algorithm described in Singh and Singh (2008). Contours enclosing 99% of the total spectro-temporal modulation power for the respective speech modulation spectra were constructed and articulation space was estimated by counting the number of distinct spectro-temporal modulations enclosed within the contour.

#### Speech modulation spectrum—a novel metric to characterize articulatory features

**Figure 1:**
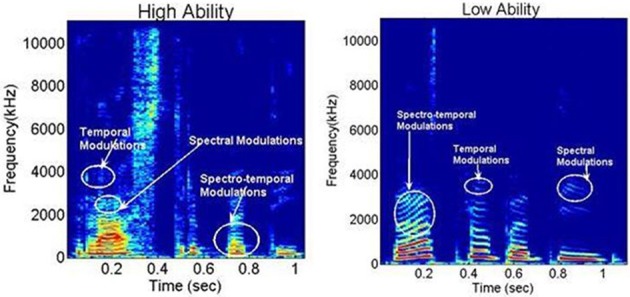
**Representative spectrograms from the High and Low Ability groups, for the Hindi word /Rashtrapati/ (meaning “president”), demonstrating spectral, temporal, and spectro-temporal modulations.** X-axis: time in ms; Y-axis spectral frequency in kHz. Notice the differences in energy distribution in the spectro-temporal modulations around 0.3 s time between the two groups.

Speech is a signal that involves processing at multiple time scales (Rosen, 1992). It is therefore proposed that articulatory features of spoken language require the sensori-motor integration of articulatory gestures at different time scales. Singh and Singh (2008) developed a novel spectral analysis technique, called SMS to study the organization of such articulatory gestures as a metric of speech motor skills. The first step of this analysis involves using speech samples from each participant to calculate a spectrogram. The spectrogram is a time-frequency representation of the speech signal and offers a visual display of fluctuations in frequency and time (see Figure 1), described respectively as spectral and temporal modulations. As shown in Figure 1, spectral modulations (ω_*f*_) are energy fluctuations across a frequency spectrum at particular times, whereas temporal modulations (ω_*t*_) are energy fluctuations at a particular frequency over time. Based on the rate of fluctuation, spectro-temporal modulations have been proposed to encode three articulatory features, namely (1) syllabicity or syllabic rhythm (SR) (2–10 Hz), (2) formant transitions (FT) reflecting consonant blends and transitions (20–40 Hz), and (3) place of articulation (POA) reflecting finer, rapid-scale changes in utterance (50–100 Hz).

**Figure 2:**
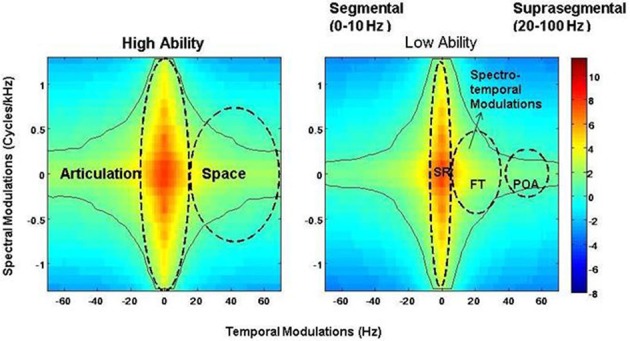
**Speech modulation spectra for the low and high ability groups, demonstrating the “articulation space” and the presence of different articulatory features.** Articulatory features are classified based on different time scales and classified thus: SR = syllabic rhythm, 0–20 Hz; red bars- FT = formant transition, 25–40 Hz; green bars – POA = place of articulation, 50–100 Hz. Color-coded bar reflects intensity of energy/power distribution. Notice differences between energy intensity at the POA time scale between the two groups.

A 2-D Fourier transform of the spectrogram yields a probability distribution of these different articulatory features and is called the SMS (Singh and Theunissen, 2003). In a typical SMS, the central region between 2 and 10 Hz carries supra-segmental information and encodes SR. The side lobes between 10 and 100 Hz carry information about segmental features. FTs are encoded between 25 and 40 Hz, and POA information is found between 50 and 100 Hz (Stevens, 1980; Tallal et al., 1985). As the modulation spectrum goes from 1 to 100 Hz, the amplitude fluctuations of a sound become faster and go from syllabic to vowel-like to plosive-like segments (Singh et al., 2007). The modulation spectrum thus plots a “language articulation map,” which depicts how energy or “power” is distributed in different articulatory features of spoken language, namely SR, FTs, and POA (see Figure 2).

Quantifiers to investigate speech features included contour areas at the three different timescales of SR, FT, and POA. The contour area is the total number of spectro-temporal modulations that encompass 99.9% of the total energy. The total contour area, therefore, is comprised of the number of spectro-temporal modulations for each articulatory feature. The contour area for each articulatory feature is the number of modulations as defined by the temporal limit for that feature—thus the contour area for SR is the number of spectro-temporal modulations between 0 and 10 Hz, for FTs the spectro-temporal modulations between 10 and 50 Hz and for place for articulation between 50 and 100 Hz. Speech Modulation Spectra for the current study were created from samples recorded in the MRI scanner during conditions A, B, and C. For more details on the method please refer to Singh and Singh (2008).

Earlier studies using the SMS (Singh and Singh, 2008; Sullivan et al., 2013) have established this method as capable of capturing differences in fine motor control in human speech. For instance, SMS analysis performed on speech samples of 160 typically developing children 4–8 years old demonstrated a developmental pattern for the three articulatory features described above: (1) adult-like patterns of syllabicity (2–10 Hz) emerged at 4 years old or earlier, (2) FTs emerged by 5 years old, and (3) POA emerged by 6–7 years old and beyond (Singh and Singh, 2008). More recently, in a study comparing toddlers with ASD, developmental delay, and typical development, this method was successful in establishing differences in speech motor function between the three groups. Additionally, significant correlations were also seen between articulatory features and language and motor ability as assessed by the behavioral measures of expressive and receptive language for the ASD group (Sullivan et al., 2013).

## Results

### Behavioral results

As a main result of the Hindi imitation native speaker ratings, we found that the scores of Hindi imitation capacity followed the shape of a Gaussian normal distribution for our 138 German participants (see also Participants and Behavioral Experiments) with 70% of subjects ranging within one SD below and above the mean (average ability). The subjects' mean score was 4.62, *SD* ± 0.99, ranging from a lowest score of 2.42 to a highest score of 7.74 on a range from 0 = min to 10 = max. The 18 Hindi native speakers who had been interspersed into the database to confuse the native raters were ranked along the first 18 places of the evaluation, scoring between 8.07 and 9.9, *SD* ± 0.6; four-five standard deviations above the mean scores of our subjects. This means they had been perfectly identified as Hindi native speakers by other Indians, a fact which controls the quality of the online rating procedure. The raters were 30 “blind,” naïve, gender balanced, (15 females) Indian native speakers living in India, performing the ratings online using earphones, indicating judgments on a 0–10 intuitive rating scale. They were paid for their services of several hours of rating time needed for a sample of 138 subjects (plus 18 native subjects). The inter-rater reliability, Cronbach's Alpha was 0.964. Originally a larger number of raters (36) performed the online ratings, but 6 raters were excluded due to their low inter-rater reliability. For more details on the Hindi speech imitations and ratings procedure also see Jilka (2009) and Reiterer et al. (2011).

The two obtained speech imitation scores of foreign language material, the Hindi and English imitation scores, also correlated significantly with each other 0.33^**^ (*p* = 0.0001). For more details on the scoring systems see also Hu et al. (2013).

### Modulation spectrum results (acoustic analyses)

**Figure 3:**
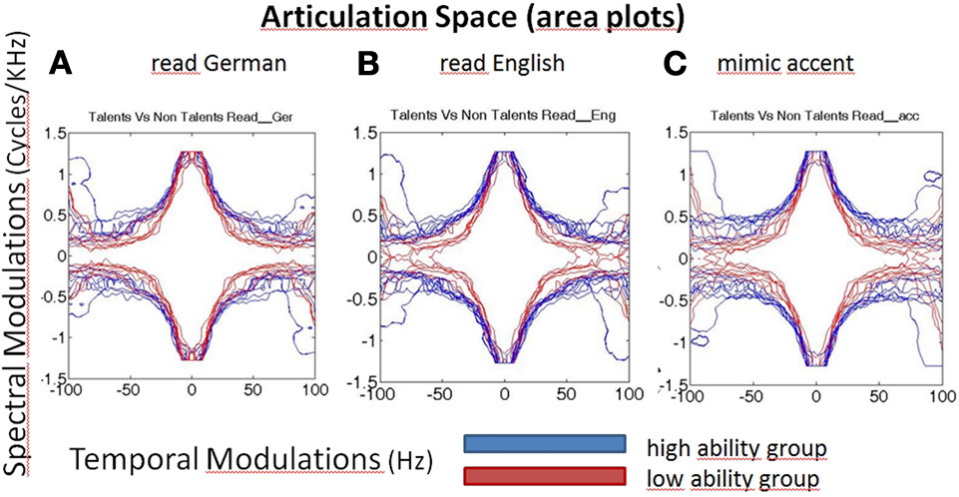
**Articulation space/area plots of the modulated speech spectrograms, characterizing speech by fluctuations of energy in different frequencies over time.** X-axis represents time (here seconds), y-axis frequency (Hertz) and the colors encode the different ability groups. Blue, high ability speech imitation group; Red, low ability speech imitation group. High ability speakers have larger articulation spaces than low ability speakers, in all three subconditions: reading German **(A)**, reading English sentences **(B)**, faking an accent (**C**, English accent in German carrier sentence).

Representative spectrograms of the low and high ability groups in Figure 1 showed differences in the energy distributions of the temporal, spectral, and spectro-temporal modulations. Differences in articulatory features were estimated using the SMS analysis, and differences across all the three conditions are displayed in Figure 2. The low ability group showed reduced energy in the place of articulation information (POA), which lies between 50 and 100 Hz, and is representative of the faster modulations. The contour areas for the different articulation spaces for the extreme high ability and low ability group (based on the Hindi imitation ability scoring) in the three conditions of our main task (Neuroimaging Analyses) reading native German sentences (A), reading L2 English sentences (B), and faking a foreign accent (C) are shown in Figure 3.

**Figure 4:**
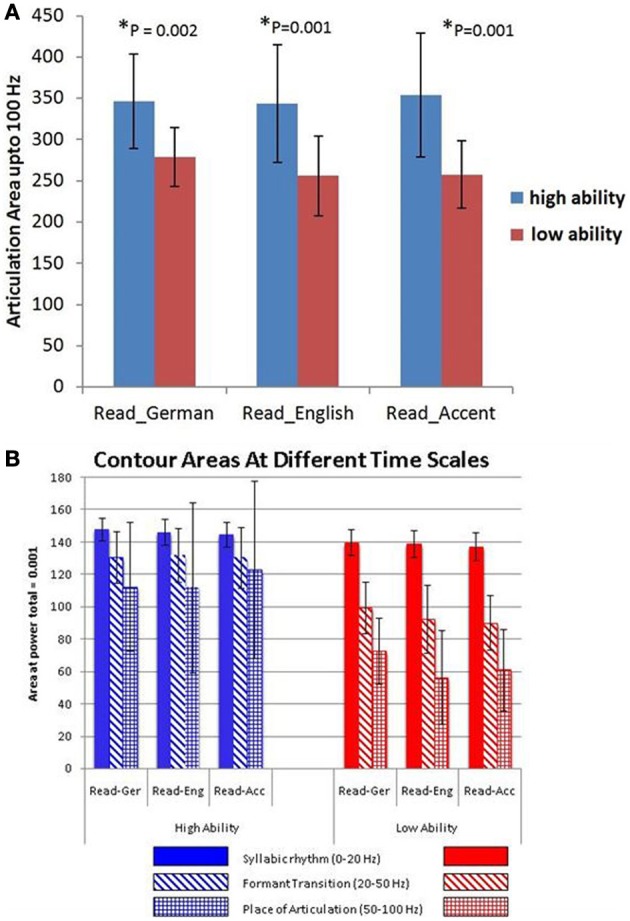
**(A)** Articulation space in numbers (area plots covering the articulation areas based on the modulation spectrum analysis) for both non-native ability groups, the German-speaking low-ability (red bars) and high-ability (blue bars) speech imitators. Bar graphs are given for the three in-scanner behavioural task conditions: Read_German, Read_English sentences and “Read_Accent” (= faking a foreign accent in mother tongue sentences, here, an English accent in German sentences). The lower panel **(B)** shows the group differences of different articulation area spaces (contour areas) in more detail according to the three different articulation time/frequency scales: blue bars - SR = syllabic rhythm, 0–20 Hz; red bars - FT = formant transition, 25–40 Hz; green bars - POA = place of articulation, 50–100 Hz.

A statistical comparison of the articulation areas between the high and low ability groups (Figure 4A) showed significant group differences (*p* = 0.002 for condition A, *p* = 0.001 for condition B, *p* = 0.001 for condition C). As shown in Figure 4A in all conditions (A, B, C), the group with high ability speakers (talents) displayed a significantly larger articulation space as compared to the low ability speakers. In condition C (faking the foreign accent) this difference was most pronounced (Figure 4A). In order to quantify the differences across various articulatory features, the area occupied by each articulatory feature for the three conditions was also calculated. As seen in Figure 4B, the high ability group shows larger area for all the three articulatory features with differences being significant for faster time scales namely FTs and POA.

**Figure 5:**
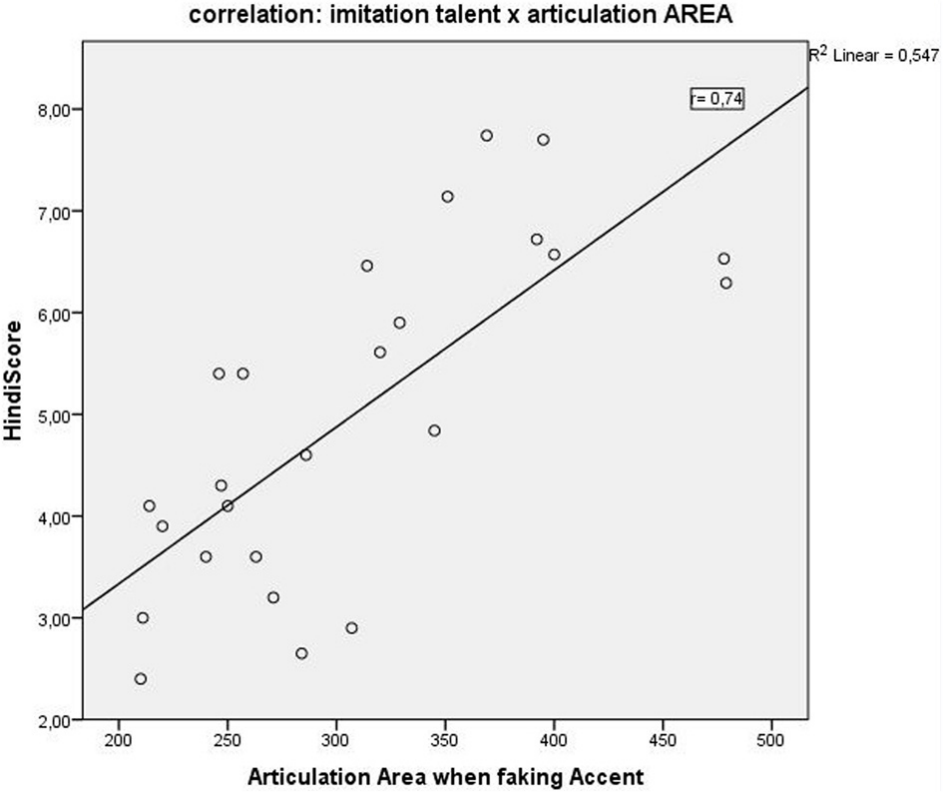
**Positive correlation (*r* = 0.7; *p* < 0.0001) between the “Hindi scores” (Hindi speech imitation ability) and the “Articulation Area or Articulation Space” as identified by our modulation spectrum analysis**.

A correlation of the articulation space area scores with the behavioral language imitation scores, showed a positive correlation in all three conditions with the highest positive correlation for condition C (faking the accent): Pearson *r* = 0.74, *p* = 0.0000^**^ (Figure 5), followed by condition B (reading L2), *r* = 0.66, *p* = 0.0002; and finally by condition A (reading German) correlated *r* = 0.62, *p* = 0.001. Articulation space also correlated with the English pronunciation ability scores, although to a lesser degree and significance level: English score correlated positively with articulation space for condition A: *r* = 0.4, *p* = 0.04; condition B: *r* = 0.5, *p* = 0.01; with condition C: *r* = 0.4, *p* = 0.02. Thus, the English imitation score correlated most strongly with condition B, the “articulation space of reading English sentences.” See also Figure 5 for the example of the correlation with condition C.

### Brain imaging (fMRI) results

#### Behavioral results of the in-scanner recordings

The microphone monitored sentences were recorded and controlled offline for omissions and error rates. We computed a global measure for the errors in sentence reading. Generally there were few errors so that it was not meaningful to split the errors into subtypes of errors. We identified: “partial omissions,” “mispronunciations,” “slips of the tongue,” “repetitions,” “self-corrections,” “hesitations”. Not a single complete omission of a sentence was observed. In all 3 sub-conditions (read German, read English, read with foreign accent) there were no statistically significant differences between the groups (*p* < 0.05; two tailed, *t* test for independent samples). Error counts do not correlate with our imitation/pronunciation ability measure, the Hindi score, only and not surprisingly, the English score correlates negatively with the error rates for reading English sentences (the higher the English pronunciation ability score, the lower the error rate, *r* = 0.6).

#### fMRI results

**Figure 6:**
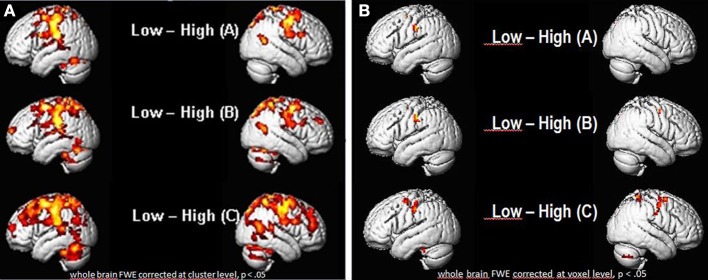
**Group vs. group comparisons: significant differential activations (low ability > high ability group) for the low ability group during sentence reading in (A) German (L1), (B) English (L2) and (C) German with fake English accent (L1Acc). (A)** FWE corrected (whole brain) for multiple comparisons at cluster level, *p* < 0.05. Cluster extent threshold *k* = 400 voxels (only clusters which exceed this number are displayed). **(B)** FWE corrected (whole brain) for multiple comparisons at voxel level, *p* < 0.05. Significant differences in activation already found for (A) reading in mother tongue, with increasing activations in speech-related areas the more complex the reading task gets (reading English sentences and German sentences with fake English accent). Activation peak observed in the left inferior parietal (IPL) cortex, supramarginal gyrus.

As seen in Figures 6A,B, there were a number of areas where the low ability group showed significantly greater fMRI (increased in intensity and extension) activation than the high ability group (Figures 6A,B; Tables 1, 2). Significant increases in activation were observed along the Rolandic fissure (central sulcus) which demarcates the motor (precentral gyrus) and somatosensory (postcentral gyrus) areas, also called the sensorimotor strip. In detail, the network activated by the low ability group comprised: (1) pre- and postcentral gyrus (bilateral), (2) inferior and superior parietal lobe, (3) basal ganglia (caudate and putamen), (4) left insula, (5) parts of the middle frontal and middle temporal gyrus and 6. cerebellum. As can be seen from Figures 6A,B, the gradual increase in difficulty during the mimicry task, is reflected in the increase of activated brain areas as the reading task becomes more complex, from German as L1 (A), over English as L2 (B) to (C) German with foreign accent. Especially when faking a foreign accent (Figure 6C; Table 2), the low ability group displays strongly increased activation within the speech-motor-relevant areas and the dorsal speech processing pathway, with peak foci around the left supramarginal gyrus/postcentral gyrus. The differences between the three conditions (A, B, C) consisted mostly in the intensity and extent of activation rather than in recruiting different areas for each condition. For example, in condition A and B (reading German and English sentences) a significant activation of only the right basal ganglia (caudate and putamen) was observed, whereas in condition C (faking the accent) the activation increased into a significant bilateral involvement of right and left basal ganglia. The overall activation increases of the low ability group in all reading tasks occurred in both hemispheres, but (based on visual inspection) seemed more pronounced within the left hemisphere. Here, the activation peak (color-coded in yellow) culminated in left inferior parietal and postcentral areas, within and around the supramarginal gyrus [peak voxel in MNI coordinates (−54, −21, 45)].

**Table 1:**
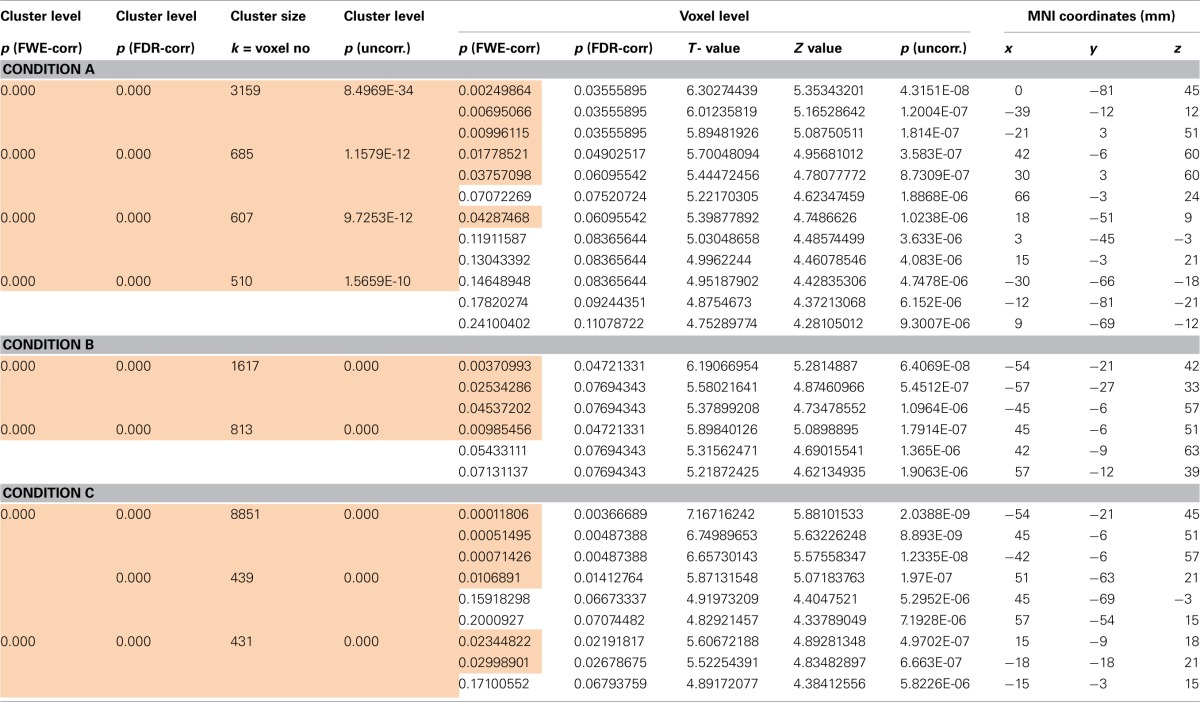
***p*-value statistics (ad Figures 6A,B) including corrections for multiple comparisons for activations at cluster and voxel level**.

Different conditions of the reading task: A (reading German sentences), B (reading English sentences), C (faking accent). Cluster size is indicated in “k”, voxel no, number of voxels. Coloured areas denote p < 0.05.

**Table 2:** **Table of the regions involved in the reading task for which the details of activations are provided in Table 1**.

**Tag**	**Location**	**Peak MNI coordinates**					**Cluster level**	
		***x***	***y***	***z***	***T***	***Z***		
	**Regions**						**Size = *k***	**P FWE**
							***k* = Number of voxels**	**Probability level**
**IMITATION TASK, CLUSTERS FOR MAIN EFFECT FOR CONDITION A (READ GERMAN SENTENCES): LOW vs. HIGH**								
1	Left precuneus, Left insula	0	−81	45	6.30	5.35	3159	0.000
2	Right SFG, Right precentral gyrus	42	−6	60	5.70	4.96	685	0.000
3	Right postcentral gyrus	39	−45	63	5.53	4.84	130	0.000
4	Left cerebellum	−27	−39	−30	5.34	4.71	200	0.000
**IMITATION TASK, CLUSTERS FOR MAIN EFFECT FOR CONDITION B (READ ENGLISH SENTENCES): LOW vs. HIGH**								
1	Left IPL, Supramarginal gyrus	−54	−21	42	6.19	5.28	1617	0.000
2	Right MFG, Right precentral g.	45	−6	51	5.90	5.09	813	0.000
3	Right calcarine	18	−51	9	5.30	4.68	309	0.000
**IMITATION TASK, CLUSTERS FOR MAIN EFFECT FOR CONDITION C (FAKING ACCENT): LOW vs. HIGH**								
1	Left IPL, Postcentral gyrus,	−54	−21	45	7.17	5.88	8851	0.000
2	Right MTG	51	−63	21	5.87	5.07	439	0.000
3	Right caudate, Left caudate	15	−9	18	5.61	4.89	431	0.000

Conditions from top to bottom: A (read German) B (read English), C (read with fake accent). Peak activation clusters for main effect of group: low vs. high. Abbreviations: IPL, inferior parietal lobe, MFG, middle frontal gyrus, MTG, middle temporal gyrus, SFG, superior frontal gyrus. Cluster size is indicated in “k”, number of voxels.

**Figure 7:**
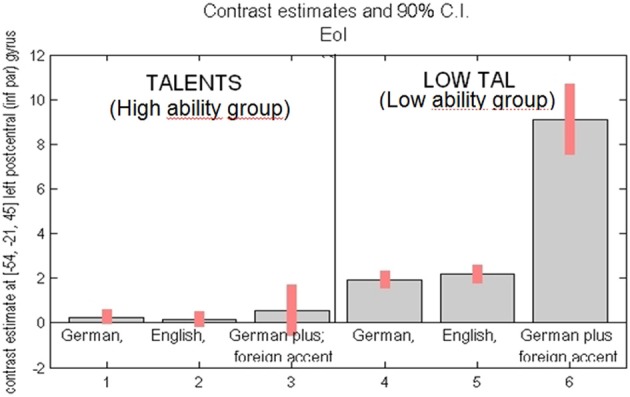
**Activation intensity as given by percent BOLD (blood oxygen level dependent) signal change (y-axis) in the peak activated area [= left inferior parietal cortex, voxel coordinates, MNI: (−54, −21, 45)].** Left side: high ability group (“talents”) for task reading sentences in (1) German (L1), (2) English (L2), (3) German with fake Engl. accent (L1Acc). Right side: low ability group (“low tal”) for the same task, reading in (4) German (L1), (5) English (L2), (6) German with fake Engl. accent (L1Acc). Most significant group difference found for reading German with foreign accent (compare 3 vs. 6).

To compare the intensity of the fMRI signal change between the groups, the highest (peak) activated voxel of the left supramarginal gyrus was chosen as ROI. The second reason for choosing this ROI was based on the hypothesis that the left supramarginal gyrus is an important hub area for sensorimotor integration (Lopez-Barroso et al., 2011; Rauschecker, 2011; Grabski et al., 2013). According to this ROI group differences in intensity of activation were estimated (see Figure 7). Within the high ability group only, the differences between the conditions were not significant. Significantly higher signal intensity was observed for the low ability (as compared to the high ability group) in all three conditions, but most strikingly in the condition where participants were required to fake a foreign accent. Thus, in the low ability group, the “big gap” occurs between normal sentence reading and faking a foreign accent.

**Figure 8:**
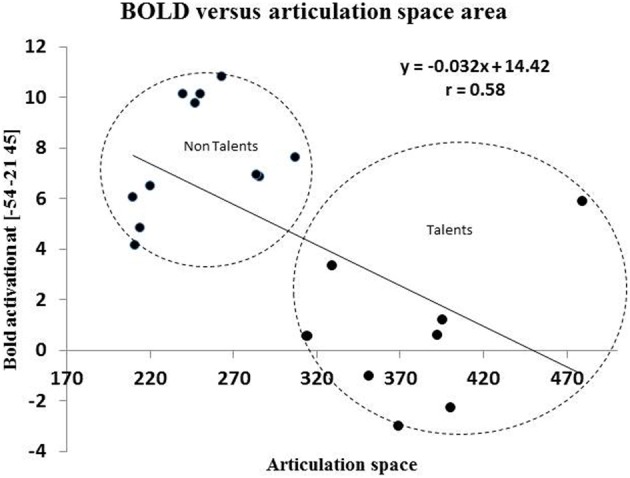
**This figures shows the correlation between BOLD activation of peak voxel [−54, −21, 45] in the left inferior parietal cortex with the area spaces of the “Articulation Space” according to modulation spectrum analysis**.

Brain behavior correlations: Since peak BOLD activity in the supramarginal gyrus and the area of the articulation space varied significantly across groups, articulation space was used as covariate in the analysis. A whole brain correlation was carried out and it was found that articulation space area correlated with activity in the supramarginal gyrus (Figure 8). As shown in Figure 8, a significant correlation was found between activity in the SMG and area of the articulation space.

## Discussion and conclusion

We compare for the first time a combination of neural and acoustic features in individuals with high and low abilities in the speech-motor imitation domain. We found significant differences between groups of high and low ability speech imitators in terms of brain imaging as well as biometric acoustic analyses, as differentiated by the task of imitating foreign accented speech. Based on our findings we propose that it is possible to characterize speech imitation talent from purely biometric data. We also provide behavioral and neuroimaging evidence that the task of faking accents from memory, which requires phonological awareness at both segmental and suprasegmental levels, is an effective discriminant of individual differences in pronunciation aptitude, rooting partly in decisive differences in auditory long term memory performance. Some individuals seem to have good access, storage and retrieval capacity of auditory episodic events, possibly stored as exemplars in their memory. Thus, they possess detailed phonetic representation and phonemic awareness of how to reproduce the typical accent of foreign-sounding speech. The auditory phonemic imagination capacity of our good speakers might also be dependent on their well-developed oro-motor system and good articulation capacity, which we tested. Those with lower awareness for foreign accented speech seem to have less successful memorization strategies perhaps due to a lower articulation capacity in the first place. Recent research supports the view that participation of the oro-motor system may be essential for laying down the memory of speech sounds and that speech and auditory memory are heavily intertwined by their coevolution (Schulze et al., 2012). Likewise, mapping sound to articulation is a process which is ascribed to partly overlapping, heavily interconnected brain areas with one hub area being located in the left parietal cortex (culminating in the supramarginal gyrus) and another hub being related to the pre-motor areas, the whole loop being referred to very often as the “dorsal” route of language processing (Saur et al., 2008; Lopez-Barroso et al., 2011; Grabski et al., 2013). We found this dorsal route significantly more activated or “taxed” in our low ability foreign accent imitators.

Our brain imaging results confirmed our initial hypothesis that low ability speakers activate speech production areas more than high ability speakers (compare Figures 6–8) and suggest that this reflects the mechanism of neural or cortical efficiency (compare Reiterer et al., 2005a,b, 2011). We found significantly different activations in areas well established to speech production, proposed by some researchers as “minimal speech production network” (Bohland and Guenther, 2006) or more generally articulation/speech production network (Ackermann and Riecker, 2004, 2010; Ackermann, 2008; Moser et al., 2009; Ackermann and Ziegler, 2010; Grabski et al., 2012; Ziegler and Ackermann, 2013). Group differences occurred primarily within the pre-and postcentral gyrus (sensory motor cortex), the left inferior and superior parietal cortex—which included the area of maximum difference—the left supramarginal gyrus, the basal ganglia system (caudate and putamen bilaterally), cerebellum, parts of the middle frontal and middle temporal gyrus, inferior frontal gyrus (Broca, pars opercularis), and the left insula.

The most significant group difference (see Figure 7), was obtained in the left inferior parietal lobe (IPL), and left supramarginal gyrus. The left IPL has been claimed to integrate aspects of speech perception and production, phonological representations, working memory store, multilingual language learning and reading, amongst others. (Rauschecker, 2011; Della Rosa et al., 2013). Recent neurobiological language processing models claim and find converging evidence for a left auditory dorsal processing stream which combines auditory speech representations in the auditory cortex with articulatory representations in the motor system by sensorimotor interaction primarily converging in the supramarginal gyrus (Lopez-Barroso et al., 2011; Rauschecker, 2011; Grabski et al., 2013). We find that low ability speech imitators highly activate this area when trying to fake a foreign accent and view this increase of activation vs. the high ability group as a sign of a compensation mechanism and use of more global workspace in these areas constituting the articulatory interface in the left IPL within the dorsal stream.

The left supramarginal gyrus/left IPL has furthermore long been associated with the phonological loop component of verbal working memory and implicated in the phonological underpinnings of reading in both native (Graves et al., 2010) and second language (Das et al., 2010). Interestingly, in a recent fMRI study on verbal working memory (Kirschen et al., 2010) a similar location of brain activation was described as a cerebral correlate of auditory and modality independent verbal working memory. Part of the explanation why the low ability group had significantly more activation particularly in the left IPL could lie in their generally weaker auditory working memory—as also strongly supported by our earlier findings (Reiterer et al., 2011). Auditory working memory correlated most significantly with the Hindi imitation ability score, i.e., the better their imitation ability of Hindi sentences, the higher their digit span of recalling numbers and non-word syllables. These correlational data with working memory were published in Reiterer et al. (2011) and Hu et al. (2013). The common ground for working memory as a trigger for aptitude in second language acquisition has long been established (Baddeley et al., 1998). It is well documented in the literature that (auditory) working memory plays a crucial role in L2 language production (Adams and Gathercole, 1995; Acheson and MacDonald, 2009; Slevc, 2011). A recent study investigating 95 school children (Andersson, 2010) re-affirmed that L2 learning success and native language processing can be predicted by working memory capacity, and that there is a strong association between L1 and L2 processing capacity, which suggests that working memory and general language aptitude are mediating mechanisms or common sources. Recent EEG studies have also shown similar results in that native monolinguals (English)—who are believed to exhibit linguistic uniformity—could be differentiated by their event related potential responses (EEG/ERP) to simple phrase structure violations into high and low L1 performers (Pakulak and Neville, 2010). In our present study, we found similar results for individual differences in brain activation even in mother tongue reading (Figure 6). We thus hypothesize that L1 and L2 language abilities are similar to each other and draw on common grounds of general language aptitude (Reiterer et al., 2005a,b, 2011).

Our SMS analysis results suggest that skilled accent imitators have a larger articulation space than poor imitators. While this is the first time this method was applied to individual differences in speech imitation ability in linguistically normal individuals, a recent study (Sullivan et al., 2013) showed that the SMS could be used to demonstrate differences in speech motor ability in a subgroup of children with autism with poor verbal skills. The current study provides additional support for the use of the SMS as metric for investigating and discriminating speech motor skills. Our working hypothesis suggests that this extension in articulation space in the high ability group might provide access to a larger repertoire of sounds, which in turn could possibly provide skilled imitators greater flexibility in pronunciation. This also provides support to our hypothesis that late but highly talented L2 speakers who are good at accent imitation, keep their phonetic categories more flexible and open for being exposed to new sounds without confining their articulatory repertoire to the mother tongue speech sound processing schemes. Neurocognitive flexibility as a determinant of language talent was proposed two decades ago by researchers examining exceptionally gifted language learners (Schneidermann and Desmarais, 1988). Recent evidence from studies with infants' phonetic language learning development confirms these earlier postulates of neurocognitive flexibility. In a series of ERP and behavioral experiments on phonetic discrimination ability in infants, Kuhl et al. (2008) showed that individual differences in phonetic discriminative behavior at an early age could predict later language capacities in L1 development. Those children who at 7 months showed better phonetic discrimination rates of native language speech contrasts, also showed faster advancement in their L1 as measured at 30 months. Those children were thus better tuned toward their L1. In contrast, the children who displayed better phonetic discrimination performance of non-native contrasts at 7 months were also slower L1 developers. The authors concluded that the brains of the slower L1 development group had remained more in the initial, more immature state, which makes them more open toward new foreign sounds but thus less neurally committed to native language speech patterns.

Our behavioral and computational acoustic data point into the same direction. There is higher neuro-cognitive flexibility, reflected by higher articulatory flexibility in the group of the more talented speech imitators. Since the Hindi imitation task did not involve any pre-existing experience with the language, we assume that some individuals must still possess this openness to build new phonetic categories on an *ad-hoc* basis (e.g., by storing exemplars through memory-based learning), and not rely on pre-experienced, entrenched categories (rule-based learning). If transfer/interference from L1 had taken place, their speech output would not have been rated as being close to the native speaker range of Hindi speakers.

With respect to phonological theories of second language learning, critical periods, and interference from L1 our speech imitation data point to a “variable” view. It is difficult to confer an all-or-nothing theory like “all second language speakers show transfer or interference from L1,” or “all late second language learners cannot phonetically imitate native-like speech because they are beyond an assumed critical period.” Instead the emerging picture is differentiated or gradual and could be summarized by saying “it depends on.” Our high ability phonetic imitators produced speech production samples similar to the native speakers of the language as reflected in the native speaker judgments. However, the low ability group was rated significantly different (very low) by native judges. This means that the world of second language speakers is not best described by a unifying theory according to which all speakers act according to either one or another theory, but seem to follow graded distributions of the form “some speakers contradict traditional theories,” “some speakers highly corroborate traditional theories,” and “many speakers are in transitional positions.” Thus, for our high ability speakers we can refute a critical period for phonetic learning and propose instead a successful mastery of native speech where interference from L1 is not at work. Phonetic interference phenomena from L1 do exist, but they depend on the individual level of phonetic aptitude.

## Conclusion

Our data provide strong evidence that speaker-related individual differences in speech imitation ability/aptitude play a decisive role in speech production, being similarly expressed for L1 and L2, and can be visualized and quantified by various biometric methods of signal analysis. Regarding speech imitation ability on a neurometric scale, we confirmed the theory of cortical processing effort by showing that both greater intensity and extension in activation of cortico-subcortical speech relevant areas can be shown as a function of speech imitation ability even in the case of the mother tongue. Poorer skills were associated with greater consumption of neural workspace. Focal area of the statistical group differences was the left supramarigal gyrus, a hub area in sensorimotor integration, along the dorsal language processing stream. With regard to the newly applied acoustic measures of speech output, we found a larger articulation space to be a marker of high ability in L2 speech imitation skills. Further research is needed to refine the acoustic parameters for discriminating differences in proficiency and ability of the speech imitation skills. We further showed that faking or imitating foreign accents is a valuable task in differentiating speakers with different levels of speech sound imitation and pronunciation ability. It remains to be clarified exactly why faking accents manifests such a considerable obstacle for speakers with low mimicking skills. We conclude from our present experiment that speech-motor flexibility, neurocognitive flexibility, working memory, and auditory long term memory may be amongst the predictors of speech imitation capacities, but the picture is surely more complex and multi-causal. Future research needs to clarify the role of auditory and sensory-motor memory in relation to other factors (e.g., musicality, personality, early experience with registers, dialects, languages) more precisely. However, it becomes clear that these individual differences visible in mother tongue as well as foreign speech processing do exist, are detectable, even predictable to a certain degree of specificity, by neurometric and audiometric means of signal analysis.

### Conflict of interest statement

The authors declare that the research was conducted in the absence of any commercial or financial relationships that could be construed as a potential conflict of interest.
